# Neurotrophin System Alterations Associated with Neurotoxicity Accompanied by Carotid Artery Diseases—A Systematic Review

**DOI:** 10.3390/ijms27062817

**Published:** 2026-03-20

**Authors:** Jovan Milosavljevic, Marina Mitrovic, Dragica Selakovic, Davor Kumburovic, Miodrag Sreckovic, Suzana Randjelovic, Sara Rosic, Miljan Cpajak, Nemanja Jovicic, Gvozden Rosic

**Affiliations:** 1 Department of Physiology, Faculty of Medical Sciences, University of Kragujevac, 34000 Kragujevac, Serbia; jovan.milosavljevic1997@gmail.com (J.M.); davor.kumburovic@gmail.com (D.K.); rosicsara@gmail.com (S.R.); miljancpajak@yahoo.com (M.C.); grosic@fmn.kg.ac.rs (G.R.); 2 Department of Medical Biochemistry, Faculty of Medical Sciences, University of Kragujevac, 34000 Kragujevac, Serbia; mitrovicmarina34@gmail.com; 3 Department of Internal Medicine, Faculty of Medical Sciences, University of Kragujevac, 34000 Kragujevac, Serbia; sreckovic7@gmail.com; 4 Clinic of Cardiology, University Clinical Center Kragujevac, 34000 Kragujevac, Serbia; 5 Department of Emergency Medicine, University Clinical Center Kragujevac, 34000 Kragujevac, Serbia; suzanarandjelovic25@gmail.com; 6 Department of Histology and Embryology, Faculty of Medical Sciences, University of Kragujevac, 34000 Kragujevac, Serbia; nemanjajovicic.kg@gmail.com

**Keywords:** neurotrophins, brain-derived neurotrophic factor—BDNF, neurotoxicity, carotid arteries disease—CAD

## Abstract

According to neuropsychiatric sequelae for cardiovascular pathology, carotid artery disease (CAD) represents a significant medical, social, and economic burden. Numerous efforts have been made to define reliable markers that can reflect the principal pathological event and the effect of employed therapeutic protocols, prognoses, and clinical outcomes of CAD. However, the potential role of the neurotrophin (NT) system has not yet been confirmed. This narrative review was conducted following a literature search of PubMed, which included all studies on NT system elements and CAD published over the last two decades, encompassing both animal and clinical investigations, regarding the potential use of NT system elements as biomarkers for neurotoxicity manifestations and therapeutic effectiveness in CAD. Still, the analysis presented in this review is not sufficient to reveal whether NT system elements can be considered as exploratory or standard biomarkers for the evaluation of CAD. Further research is essential to elucidate this dilemma.

## 1. Introduction

Cerebrovascular ischemia is among the most important causes of cardiovascular morbidity and mortality in developed countries and one of the leading causes of social isolation, with a remarkable socio-economic burden on the healthcare system. Carotid artery disease (CAD) represents a leading cause of ischemic stroke [1]. Prevalence is closely linked to the aging process, showing a marked increase in incidence as the population grows older [2]. With the aging global demographic, addressing this condition is not only a clinical necessity but also a socio-economic need. It is estimated that CAD is responsible for 7% to 25% of all ischemic strokes, making it a critical target for preventive and secondary medicine [3,4].

Incidence of carotid artery stenosis (CAS) is increasing due to the widespread popularity of high-fat and high-salt diets, sedentary lifestyles, and the increasing age of the population. Attention should be drawn to the fact that individuals with atherosclerotic CAS can have a prevalence of atherosclerotic coronary artery disease as high as 50 to 75% [5]. The prevalence of carotid stenosis in the general population is low (3%), and routine screening for carotid stenosis is not recommended in adults [6].

Precise classification of the degree of CAS primarily relies on radiological parameters obtained via duplex ultrasound, CT angiography (CTA), or magnetic resonance angiography (MRA). The gold standard in diagnostics is the NASCET method (North American Symptomatic Carotid Endarterectomy Trial), which calculates the percentage of stenosis by comparing the narrowest lumen to the distal healthy segment of the artery. It is important to differentiate between the two main methods for calculating the percentage of CAS, as they provide different numerical values for the same physical narrowing.

NASCET (North American method) uses the diameter of the distal internal carotid artery (ICA) as the denominator. This method is the standard in most modern clinical guidelines.

ECST (European method) estimates the original (theoretical) diameter of the artery at the site of the narrowing itself. Because of the wider denominator, the ECST percentage is always higher than the NASCET percentage (e.g., a NASCET percentage of 50% corresponds to an ECST value of approximately 70%) [7].

Beyond the degree of narrowing, the following factors are also important for clinical evaluation:

Plaque morphology: A distinction is made between stable plaques (calcified, smooth) and vulnerable plaques (containing a soft lipid core, intraplaque hemorrhage, or ulceration). The latter are associated with a much higher risk of embolization, even in cases of lower-grade stenosis.

Anatomical type (Weibel–Fields classification): Tortuosity (T-type), kinking (K-type), and coiling (C-type) can affect local hemodynamics and contribute to neurotoxicity independent of atherosclerotic narrowing [8].

### 1.1. Clinical Manifestations and Classification

It is important to emphasize that CAD manifests through a highly heterogeneous spectrum of neurological and neuropsychiatric symptoms, as presented in Table 1. The clinical presentation is not limited to episodic ischemic events. It encompasses a wide array of phenotypes driven by chronic cerebral hypoperfusion (CCH), microembolization, and impaired regional functional connectivity. Clinical presentation includes the following:

- Acute ischemic syndromes: clinical presentations of transient ischemic attack (TIA) and stroke, including contralateral hemiparesis, dysphasia, and ipsilateral blindness (amaurosis fugax);
- Cognitive impairment: deficits and decline in executive function, attention, working memory, and learning/recall are frequently observed;
- Neuropsychiatric and behavioral symptoms: a high prevalence of anxiety and vascular depression is registered, often correlating with the severity of white matter lesions;
- Atypical and hemodynamic symptoms: patients frequently report diverse symptoms such as generalized fatigue, syncope, “dizziness”, and “limb shaking” which reflect hemodynamic compromise rather than just embolization.

**Table 1:** Summary of the clinical and radiological classification of carotid artery diseases.

Syndrome/Grade	Clinical Diagnostic Criteria	Radiological/Stenosis Criteria
Transient ischemic attack (TIA)	Symptoms resolve completely within 24 h	No evidence of infarction on CNS imaging
Ischemic stroke	Symptoms persist > 24 h	Presence of infarction on CT/MRI
Mild stenosis	Asymptomatic in most cases	<50% diameter reduction
Moderate stenosis	Potential for cognitive decline	50–69% diameter reduction
Severe stenosis	High risk of acute events	70–99% diameter reduction
Total occlusion	Variable (collateral-dependent)	100% diameter reduction

High-grade CAS is recognized as an independent risk factor for vascular cognitive impairment, even in the absence of a clinical stroke. Studies have specifically identified deficits in executive function, attention, and learning capacity as the hallmark cognitive profile for these patients. CCH resulting from carotid stenosis leads to quantitative and qualitative defects in adult hippocampal neurogenesis (AHN), including reduced neuron counts and aberrant structural morphology. Latest results from the CREST-2 sub-study (2026) demonstrate that while revascularization (CEA/CAS) effectively reduces stroke risk, it does not show improvements in cognitive trajectories compared to intensive medical management alone [9].

It is crucial to understand that cognitive decline in patients with carotid disorders is increasingly viewed as a multifactorial process. Beyond large-vessel mechanical obstruction and hypoperfusion, the progression toward vascular cognitive impairment involves a complex synergy of chronic neuroinflammation, small-vessel disease (SVD), blood–brain barrier (BBB) disruption, and neurodegeneration [10,11]. CCH is a trigger for a molecular injury cascade that leads to the breakdown of the BBB, which is considered the primary mechanism in the development of SVD. It is also responsible for the subsequent appearance of white matter hyperintensities. This disruption facilitates the leakage of toxic substances and the activation of neuroinflammatory pathways, characterized by elevated pro-inflammatory cytokines such as TNF-α and IL-1β, which exacerbate astrocyte dysfunction. Since these various cellular pathways are responsible for cognitive deterioration, mechanical revascularization alone may be insufficient to address the patient’s holistic neurological status [12].

### 1.2. Laboratory Markers for Clinical Evaluation and Therapeutic Effectiveness

**Figure 1:**
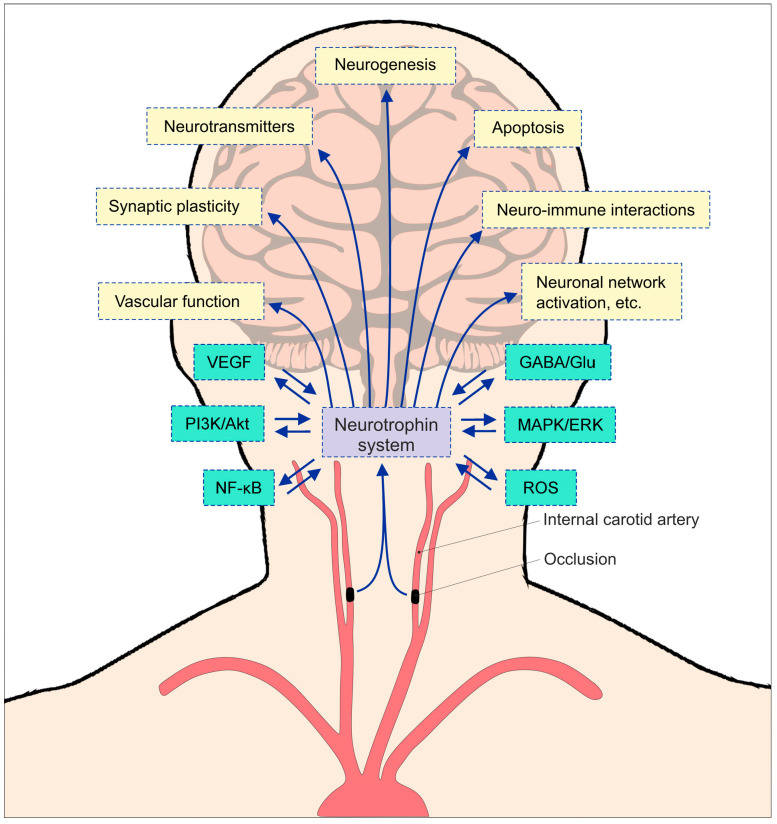
An overview of NT system effects with downstream mechanisms accompanied by CAD.

The evaluation of carotid artery disease is shifting toward the assessment of molecular and laboratory-based indicators that correlate with active neurotoxicity and real-time therapeutic responses. Current clinical practice depends heavily on systemic metabolic and inflammatory markers, yet these possess numerous limitations that hinder effective monitoring of the disease course. Traditional markers such as total homocysteine, lipoproteins (LDL, HDL), and C-reactive protein (CRP) reflect generalized systemic atherosclerosis and cardiovascular risk rather than the specific trophic state or injury degree of the brain tissue downstream of the carotid lesion. Baseline lipid levels often poorly correlate with the vulnerability or stability of the carotid plaque. Furthermore, the systemic inflammation marker standard hs-CRP has shown inconsistent associations with cognitive impairment in a group of patients with asymptomatic stenosis, suggesting that it is not the primary driver of functional impairment. These markers are used mostly for risk stratification; they do not provide adequate insight into the success of revascularization [13]. Bearing in mind the multifactorial nature of CAD, where mechanical flow, inflammation, and neurotrophic failure interplay, there is an urgent need for novel markers. They are needed to reflect the “vascular reserve” and the brain’s endogenous capacity to repair itself under chronic stress. Their role is also to track and show real-time clinical correlation of revascularization success. Markers like neurofilament light chain (NfL) (axonal damage) and brain-derived neurotrophic factor (BDNF) (synaptic plasticity) are emerging as essential tools to bridge the gap between mechanical imaging and clinical neurological outcomes. NTs (Figure 1) are also used for refining risk stratification and detecting early-stage neurotoxicity that traditional markers fail to recognize. Clinical studies have shown that in patients with significant carotid stenosis, defined as >70% reduction in lumen diameter, there are markedly lower serum BDNF levels compared to healthy controls. This depletion suggests a compromised neuroprotective environment prior to intervention [14]. A key clinical advantage of NT system tracking is its dynamic response to treatment. Successful revascularization through CAS has been shown to induce a rapid, significant increase in serum BDNF within 24 h [15]. This rise serves as a “cerebral correlate” of successful reperfusion and successful tissue repair, analogous to how troponin is used to monitor cardiac interventions. Tracking these levels provides real-time information that traditional markers, which focus on systemic inflammation or lipid profiles, fail to recognize. Latest studies also propose the use of NTs to refine risk stratification, in synergy with NfL, a marker of axonal damage. Moreover, the neurotrophic signaling is essential for maintaining the integrity of the BBB. BDNF/TrkB signaling decreases vascular permeability by inhibiting MMP-9 secretion and reducing neuroinflammation [16,17].

The aim of this narrative review is to present a comprehensive overview of the evidence gathered over the last two decades regarding the potential usage of NT system elements as biomarkers for neurotoxicity and therapeutic effectiveness in CAD.

### 1.3. NT System and Cardiovascular System

NTs belong to a group of extracellular proteins that function as growth factors to promote neuronal growth, survival, and development across the CNS and PNS. The mammalian family of NTs includes four related proteins: BDNF, NGF, NT-3, and NT-4/5. These mature NTs are released by both neuronal and non-neuronal cells, including oligodendrocytes, astrocytes, and endothelial cells, and exert their physiological effects by binding to one or more of the three tropomyosin-related kinase (Trk) receptors, TrkA, TrkB, and TrkC. NGF has a strong preference for TrkA, BDNF, and NT-4/5 and mainly activates TrkB, while NT-3 can stimulate all three Trk receptors [18]. Initially, NTs are synthesized as larger precursors, called proNTs (pro-NTs). Proteolytic cleavage converts pro-NTs into their mature-form NTs, shifting their receptor preferences. In contrast to the actions of the NT/Trk receptor complex, unprocessed pro-NTs preferentially bind to the p75 NT receptor (p75NTR), generating a signaling complex that frequently lowers synaptic activity and promotes apoptosis, particularly when Trk receptors are not co-expressed [19]. Consequently, the balance between pro-NT cleavage and Trk versus p75NTR signaling presents a key regulatory point that determines whether NTs support neuronal survival or trigger cell death [20].

Although NTs are known to be highly expressed in the CNS and PNS, new research suggests that they are also highly expressed and play significant roles outside of neurons, in non-neuronal tissues like the heart, lungs, blood vessels, immune cells, and endocrine organs [21]. Numerous studies indicated that NTs and their Trk receptors are involved in lung cell function and development, as well as neural connections, particularly via the BDNF/TrkB pathway. A recent study emphasized the role of BDNF and NT-4 in the development and innervation of lung smooth muscle cells, as demonstrated by the reduced axonal branching and axon length in BDNF knockout embryos, without affecting lung morphogenesis [22]. Accordingly, NTs may be involved in the physiology and pathophysiology of airway illnesses, neonatal lung diseases, lung fibrosis, allergies and inflammatory diseases, and lung cancer [23]. BDNF and TrkB are highly expressed in the GI tract and regulate intestinal motility, immunity, secretion, mucosal integrity, and sensation. Dysregulated BDNF/TrkB signaling is linked to GI disorders like inflammatory bowel disease [24]. Interestingly, gut microbiota may produce and detect neurochemical compounds like short-chain fatty acids (SCFAs), which contribute to neurogenesis and NT synthesis. SCFAs like butyrate enhance BDNF expression, while intestinal dysbiosis decreases it, which may impair synaptic plasticity and brain development [25,26]. BDNF can also be produced by T-cells, B-cells, and monocytes in peripheral blood and inflammatory brain lesions like multiple sclerosis (MS), as well as by microglia and astrocytes in the CNS, which may modulate inflammatory responses by elevating oligodendrocyte lineage cells and myelin proteins, rescuing injured or degenerating neurons, and stimulating axonal outgrowth [27].

According to numerous recent studies, NTs may be involved in the regulation of vascular tone, angiogenesis, inflammation, and myocardium remodeling in the CVS [28]. NTs, such as NGF and BDNF, are expressed at moderate levels in the healthy human heart, mainly in cardiomyocytes, endothelial cells, and vascular smooth muscle cells. Nevertheless, their expressions are greatly altered in cardiovascular injury-sensitive cardiomyocytes and heart tissue [29]. One of the earliest studies conducted by Ebendal and colleagues in 1979 found that heart explants support sensory neuron neurite outgrowth in vitro, demonstrating that heart cells could secrete NTs [30]. Neural crest-derived sensory, sympathetic, and parasympathetic neurons regulate heart rate and contractility, which could require NTs throughout development and survival [31]. Indeed, NTs are involved in the regulation of cardiac nerve outgrowth, axonal arborization, and trophic support in the sympathetic nervous system [32]. In the early stages of cardiovascular development, NTs and Trk receptors are important for the development of the heart and the regulation of vascular growth. BDNF and its receptor TrkB are expressed in endothelial cells of coronary arteries, and the signaling of the BDNF/TrkB pathway makes an important contribution to the development of capillary growth and the formation of the endothelium in the heart tissue during late gestation. In addition, increased expression of NGF in the vascular system can trigger the development of sympathetic hyperinnervation, which results in hypertension [33]. In view of this, many earlier studies showed that BDNF/TrkB signaling played an essential role in the regulation of pericytes and smooth muscle cells (SMCs) in heart development, as shown by the increased capillary density in BDNF-overexpressing mice [34] and reduced blood vessel density, increased vascular permeability, and increased endothelial cell apoptosis in TrkB knockout mice [35]. In addition, the conditional deletion of TrkB in SMCs also emphasizes its importance, resulting in a significant reduction in pericyte/SMC density in the heart, which resulted in perinatal lethality [36].

In postnatal life, NTs regulate the survival of endothelial cells (ECs), cardiomyocytes, and vascular smooth muscle cells (VSMCs) and modulate angiogenesis and vasculogenesis through autocrine and paracrine mechanisms [37]. Therefore, numerous studies have suggested that BDNF/TrkB signaling plays a significant role in a variety of cardiovascular disorders [38]. Accordingly, a study discovered that BDNF deficiency together with TrkB receptor malfunction could lead to severe heart defects in mice, which included endothelial cell death and structural heart abnormalities that resulted in early postnatal death [39]. The study performed by Feng and colleagues [40] showed that the BDNF/TrkB signaling pathway is vital for the maintenance of cardiac function, as evidenced by the defective cardiac contraction and relaxation of cardiac-specific TrkB (TrkB−/−) knockout mice. Additionally, another study showed a novel function for BDNF in controlling the contractility of the heart without nervous system innervation through the truncated TrkB.T1 receptor in cardiomyocytes. Lack of TrkB.T1 impaired calcium signaling and resulted in cardiomyopathy, which suggested an autocrine or paracrine effect mediated by BDNF from cardiomyocytes [41]. Furthermore, another study showed that BDNF was involved in the autonomic nervous system regulation of adult cardiovascular functions, specifically in the regulation of heart rates through the enhancement of activity in the brainstem’s cardioinhibitory parasympathetic neurons. Mice that had low BDNF expression demonstrated higher resting heart rates due to the low activity of these neurons, which were affected by changes in neurotransmission. Moreover, BDNF infusion was able to restore the normal heart rates in BDNF-deficient mice [42]. A separate study observed that the absence of BDNF or TrkB in the adult heart resulted in an increased level of cardiac dysfunction after myocardial infarction (MI). The study concluded that BDNF from the brain exerted a protective role in myocardial remodeling after MI, suggesting that the brain–heart axis is affected by NTs [42]. It has been shown that BDNF-TrkB signaling plays a crucial role in reducing myocardial apoptosis and alleviating cardiac ischemic injury by influencing the TRPC3/6 channel, while it also enhances Bcl-2 expression and decreases caspase-3 activity, thereby inhibiting apoptosis and MI in rats [43] (Figure 1). Notably, disruptions in the brain–heart axis have been increasingly recognized as a significant factor in the onset and progression of MI [44].

Furthermore, NTs play critical roles in protection from ischemia and cardiomyocyte death induced by ischemia/reperfusion (I/R). Studies have demonstrated that the beta 2 adrenoceptor agonist and caveolin-3 increase BDNF/TrkB and cAMP/PK signaling in diabetic hearts and provide protection against acute MI/I/R damage [45], while NT-3 prevents apoptotic death through the ERK/Bim pathway and enhances angiogenesis [46]. In addition, the interaction between NGF and its receptor, TrkA, provided protection from I/R injury through the PI3K/Akt survival pathway [47]. In addition, BDNF has been shown to promote myocardial cell proliferation and protect them from ischemia and hypoxic damage [48]. Further, another study found that miR-322 plays an important role in the activation of hypoxia-induced apoptosis in neonatal murine cardiomyocytes. By downregulating miR-322 through lentiviral transduction, the study showed a protective effect against hypoxia-induced apoptosis, which was linked to the upregulation of BDNF gene expression. It also showed that BDNF silencing eliminated the protective effect of downregulation of miR-322, which indicated that the miR-322/BDNF pathway played a critical role in cardiomyocyte survival in hypoxic conditions [49].

Furthermore, another study discovered that patients with coronary atherosclerosis show higher BDNF levels in the perivascular adipose tissue that surrounds their proximal aorta compared to their internal mammary artery. There was also a simultaneous decrease in TrkB expression and an increase in inhibitors of TrkB signaling, like protein tyrosine phosphatase 1B, suggesting that the vascular BDNF signaling is decreased or lost in patients with coronary atherosclerosis [50]. The relationship between cardiovascular health and BDNF plasma levels is intricate and multifaceted. It has been found that there is a positive correlation between plasma levels of BDNF and diastolic blood pressure, although the results are conflicting since it is not clear if this association is causal in nature due to the cross-sectional study [51]. Although the elevated levels of plasma BDNF in hypertensive patients have been observed, the reduced endothelial expression of BDNF suggests that the endothelial cells are not the source of elevated plasma BDNF levels [52]. Conversely, in another study, those subjects with low plasma BDNF levels and high trans-fat intake had the highest risk of hypertension [51]. Moreover, it has been found that levels of BDNF are altered in various cardiovascular conditions, such as increased levels in atherosclerotic arteries [53,54], as well as reduced levels in metabolic syndrome [55], acute coronary syndrome [56,57], and type 2 diabetes associated with cognitive impairment [58].

The relationship between cardiovascular diseases and mood disorders is complex and influenced by NTs, particularly BDNF. Numerous studies have shown that lower BDNF expression is associated with depression, and it has also been linked to hypertension through the effects on arterial baroreceptors, endothelial nitric oxide synthase, and the renin–angiotensin system. BDNF was also discovered to predict cardiovascular disease (CVD) outcomes in various patient populations [59]. For all aforementioned reasons, changes in NT concentrations in the blood circulation and the heart may serve as recognized indicators of atherosclerosis, hypoxic–ischemic damage, heart failure, hypertrophy of the heart muscle, and endothelial dysfunction [60,61].

The neurovascular unit, consisting of neurons, glia, brain microvascular endothelial cells (BMECs), pericytes, and extracellular matrix, is a key organizational structure of the CNS that ensures the coupling of metabolic demands, synaptic activity, and cerebral blood flow [62]. Disruption of this neurovascular homeostasis has been linked to various neurological and neuropsychiatric disorders, particularly those of vascular origin, such as stroke, vascular dementia, and cognitive impairments resulting from cerebrovascular disease; this condition worsens due to the influence of vascular risk factors, including hypertension, obesity, cardiac arrhythmia, hyperactivation of the renin–angiotensin–aldosterone system, and diabetes [63,64]. In this context, NTs, including BDNF, and angiogenic growth factors, such as vascular endothelial growth factor (VEGF), are crucial players in the regulation of both physiological and pathological processes of neurovascular coupling and those that occur after injury, including vascular dementia and cognitive impairment [65,66].

Both the BDNF and VEGF signaling pathways converge on similar intracellular signaling mechanisms, such as PI3K/Akt and MAPK/ERK. These signaling mechanisms have been shown to play a crucial role in the regulation of various cellular activities, such as survival, proliferation, plasticity, and angiogenesis. This provides the basis for the explanation of the mechanisms that may be involved in the coordinated regulation of both vascular and neurological activities, which have been implicated in a wide range of physiological and pathological conditions. For instance, both BDNF and VEGF have been shown to be upregulated by neuronal activity, exercise, and antidepressants, but both have been shown to be downregulated by chronic stress, inflammation, aging, and the pathology of various neuropsychiatric and neurodegenerative disorders, such as major depressive disorder (MDD) and Alzheimer’s disease (AD) [67,68].

Additionally, BDNF and VEGF have bidirectional regulatory connections. First, BDNF can increase the secretion of VEGF. Thus, a study has shown that chondrosarcoma cells secrete more VEGF-C when exposed to BDNFs and that LEC migration and tube formation are both improved by BDNFs via VEGF-C-dependent pathways [69]. Another study has also shown that BDNF can activate the TrkB/ERK signaling pathway, which, in turn, stimulates VEGF expression and secretion in osteoblasts [70]. On the other hand, VEGF can also increase BDNF production. Through BDNF/TrkB signaling, Le and colleagues [71] showed that VEGF enhanced BDNF synthesis, Müller cell viability, and neuroprotection in diabetic retinopathy. Indeed, earlier research demonstrated that diabetic VEGFR2 knockout mice exhibited a more rapid decline in retinal BDNF levels [72]. Further, another study showed that overexpression of VEGF-C promoted meningeal lymphatic vessel formation, which was essential for tissue clearance and immune surveillance in the CNS. In a mouse model of ischemic stroke, AAV-VEGF-C increased CNS-derived fluid drainage and promoted neuroprotective signaling pathways by upregulating calcium and BDNF signaling pathways in brain cells, reducing stroke injury and improving motor performance [73]. Contrary to this, a recent study showed that no significant correlation was found between BDNF and VEGF levels and cognitive change in acute ischemic stroke. This indicates that their neuroprotective effects are influenced by factors such as inflammation, age, and comorbidities, thereby constraining their reliability as the sole indicators of cognitive recovery [74].

Clinical studies have shown that patients with different vascular diseases, including hypertension, stroke, peripheral vascular disease, coronary artery disease, ischemic heart disease, heart failure, and vascular dementia, frequently experience depression. In one of the earliest studies conducted, Alexopoulus et al. [75] presented the “vascular depression hypothesis” that summarized these findings. As a matter of fact, a growing body of recent research supports this hypothesis that depressive symptoms are associated with an increased risk of CVD-related morbidity and mortality [76]. In line with this, a study by Deyama et al. [77] illustrated the complex relationship between BDNF and VEGF signaling, revealing that the antidepressant effects of BDNF in the medial prefrontal cortex (mPFC) were inhibited by a VEGF neutralizing antibody. Additionally, neuron-specific deletion of VEGF in the mPFC inhibited BDNF’s antidepressant effect. In primary cortical neurons, BDNF stimulated the secretion of VEGF, and a VEGF-Flk-1 antagonist inhibited BDNF-induced dendritic outgrowth complexity. Interestingly, crosstalk effects are also seen, suggesting that VEGF plays a BDNF-dependent function in neural regeneration and antidepressant responses. Additionally, it has been found that treatment with quetiapine, an atypical antipsychotic, in drug-naive first-episode psychosis patients resulted in significant increases in the serum concentrations of BDNF and VEGF, which were associated with improvement in psychotic symptoms. These findings not only linked VEGF to psychosis for the first time but also suggested that BDNF and VEGF could be utilized as biomarkers to evaluate treatment efficacy [78].

Moreover, it has been found that post-stroke depression (PSD) can significantly affect the quality of life of the patients, and this is associated with reduced levels of both BDNF [79] and VEGF, and low-intensity blood flow resistance training has been shown to increase serum BDNF and VEGF levels in patients with PST by elevating the concentration of blood lactic acid in their bodies [80]. On the other hand, another study observed that PSD symptoms were associated with increased levels of VEGFA due to the increased permeability of the BBB and the reduced expression of tight junction proteins like claudin-5, which further led to neuroinflammation and increased anxiety and depression-like behaviors in mice [81].

Although the relationship between BDNF and VEGF has been widely explored in primary psychiatric illnesses such as depression, schizophrenia, and stroke, the direct evidence regarding the role of the combined action of these two factors in the neuropsychiatric symptoms of vascular dementia, small vessel disease, and chronic cerebrovascular disease is somewhat limited, and it is recently gaining significant attention [82,83,84,85,86]. Overall, the NT system is an important regulator of cardiovascular health and disease, extending much beyond its traditional function in the nervous system.

## 2. Methods

**Figure 2:**
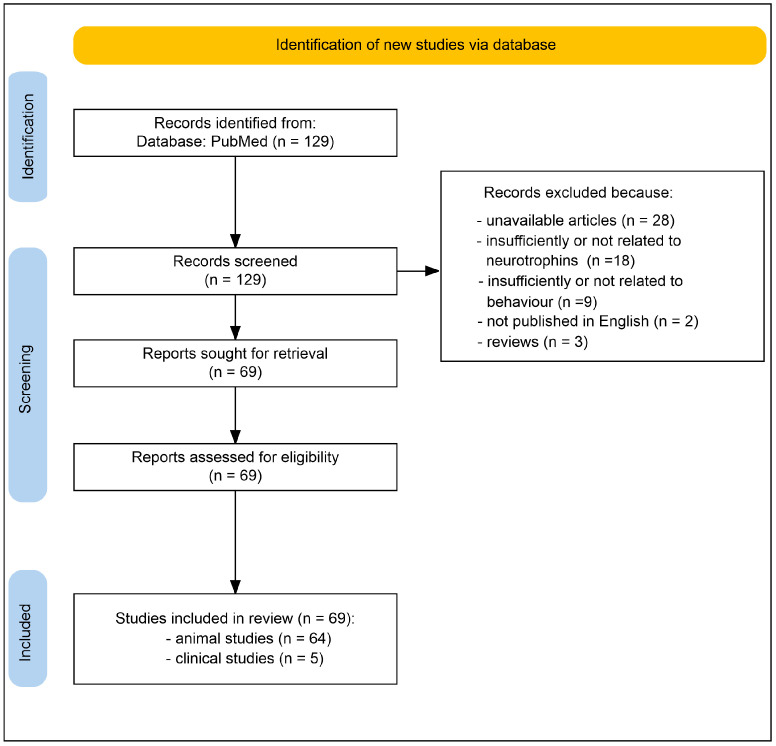
The flow diagram shows the process of screening and selecting studies.

This review was performed in accordance with the guidelines set forth by the Preferred Reporting Items for Systematic Reviews and Meta-Analyses (PRISMA) [87]. A thorough search was conducted in PubMed, covering all studies published from 1 January 2006–9 February 2026 (Figure 2). The search approach employed Boolean operators to link the following keywords: “neurotrophin” AND (“carotid stenosis” OR “carotid occlusion” OR “carotid diseases” OR “bilateral common carotid artery occlusion” OR “BCAS” OR “2VO” OR “chronic cerebral hypoperfusion”) AND (“BDNF” OR “brain-derived neurotrophic factor” OR “NGF” OR “nerve growth factor” OR “NT-3” OR “p75NTR” OR “NT-4” OR “TrkB” OR “TrkA” OR “TrkC” OR “NT” OR “neurotrophic”) AND (“behavior” OR “cognitive” OR “memory” OR “neurological” OR “psychiatric” OR “depression” OR “anxiety”). Additional records were obtained by reviewing the reference lists of the selected studies and relevant reviews. The studies considered included both animal experiments and clinical studies that specifically addressed the subject. The inclusion criteria required a comprehensive account of the molecular and laboratory-based indicators that correlate with active neurotoxicity and real-time therapeutic responses related to carotid artery diseases, as well as the mechanistic functions of components within the neurotrophic system. The exclusion criteria consisted of review articles, non-peer-reviewed publications, conference presentations, studies not pertinent to the topic, case reports lacking empirical data, and articles that were not available in English. Two reviewers performed an independent evaluation of titles and abstracts and then obtained full texts for studies that seemed potentially relevant. Any disagreements were settled through consensus among the co-authors. As other databases were not accessible to the authors due to restricted resources, this review is limited by the fact that the literature search was conducted solely on PubMed.

The final protocol was registered with the Open Science Framework on 13 February 2026 (https://osf.io/qdgv4).

## 3. Results and Discussion

### 3.1. CAD and the NT System in Clinical Trials

The carotid arteries maintain essential functions for brain health, and current clinical and preclinical research demonstrates the important role of the NT system in sustaining both vascular health and neurobiological functioning. The research shows that NT signaling alterations could lead to various CADs, providing new research possibilities that may lead to the development of new NT-based effective treatments for these serious health issues.

Smith and colleagues [88] conducted the SABPA study with a cross-sectional population cohort and showed that baseline serum BDNF was attenuated and inversely associated with indices of hypertrophic vascular remodeling, including increased vascular wall thickness and cardiometabolic risk markers (Table 2). Because BDNF was measured at a single time point, the study reflects chronic NT status rather than dynamic changes. The findings suggest that persistently low BDNF may contribute to structural vascular alterations, potentially through impaired endothelial–neuronal signaling and reduced neurovascular resilience.

In patients with advanced carotid atherosclerosis, a chronic imbalance of NTs is also evident. Yaneva-Sirakova et al. [89] noted that patients with significant carotid stenosis exhibited lower baseline levels of BDNF compared with healthy controls. Conversely, NGF levels were high, indicating a compensatory or stress-related neurotrophic response related to chronic disease. The study also demonstrated a significant increase in the levels of BDNF 24 h after carotid stenting, returning to levels comparable to those of healthy controls by one month after surgery. This pattern indicates that the restoration of cerebral perfusion rapidly normalizes neurotrophic signaling, supporting the view that circulating BDNF reflects a perfusion-dependent recovery of neuronal and neurovascular function rather than indicating irreversible neuronal injury.

The role of NTs in the context of stenting can be further explained using a double-masked randomized trial in which the application of dexmedetomidine (DEX) for 3 days after carotid stenting was carried out. In the study by Chang and colleagues [90], pre-stenting BDNF levels were comparable across treatment groups, indicating similar baseline neurotrophic status, although oscillations were observed. However, in the context of the application of DEX, it was revealed that in the 24 h and 72 h time frames following stenting, the levels of BDNF were higher in the DEX group in comparison to the placebo control, and there was a significant decrease in cerebral hyperperfusion syndrome, suggesting that augmentation of neurotrophic signaling may contribute to stabilization of neurovascular coupling and protection against reperfusion injury.

Comparable but more time-specific patterns were observed by Gao et al. [91], who observed that DEX used in carotid endarterectomy procedures elevated BDNF levels 15 min after carotid unclamping, which remained elevated 6 and 24 h postoperatively when compared to the baseline. In the placebo group, the same changes were observed. Still, no change was detected 24 h after surgery relative to the preoperative levels, reinforcing the idea that BDNF release is tightly linked to the ischemia–reperfusion transition. Sustained postoperative BDNF elevation in the DEX group was associated with attenuated neuroinflammation and faster early cognitive recovery, underscoring the functional relevance of NT modulation (Table 2).

In contrast to revascularization-based interventions, intermittent whole-body hypoxic preconditioning did not significantly alter circulating BDNF levels when measured preoperatively or postoperatively, despite activation of hypoxia-responsive pathways and reduced neuronal injury markers. This finding suggests that BDNF modulation is not a universal response to cerebral stress but rather appears to be specific to reperfusion and pharmacological neuromodulation, thereby distinguishing NT signaling from generalized hypoxic adaptation [92].

### 3.2. CAD and NT System in Preclinical Trials

Recent clinical research has begun to link NTs, particularly BDNF, to carotid artery pathologies, although human data remain limited. In contrast, studies using experimental animal models of carotid artery occlusion and the consequent development of CCH are extensive. These models provide a broad spectrum for the analysis of NT signaling in different brain regions, vascular systems, and synaptic networks during hypoperfusion and subsequent recovery. Although the animal models do not pathoanatomically replicate focal carotid stenosis, they effectively mimic the hemodynamic, metabolic, and cognitive consequences of advanced carotid disease, which are characterized by persistent cerebral hypoperfusion. Significantly, animal studies have gone beyond mere correlation to a causal relationship, demonstrating that targeted modulation of NT pathways can significantly impact synaptic plasticity, neuronal viability, and cognitive outcomes, thereby establishing NTs as active mediators in vascular cognitive impairment rather than passive disease markers.

Damodaran et al. [93] demonstrated that BDNF expression within the hippocampus was severely reduced following permanent bilateral carotid artery occlusion (PBOCCA); the levels were reduced both at 14 and 28 days postocclusion, indicating prolonged disruption of neurotrophic signaling (Table 3). Interestingly, although there were no effects on locomotor function, cognitive function was adversely affected, suggesting a stronger correlation between BDNF downregulation and cognitive impairment than with gross motor deficits in chronic cerebral ischemia.

Using the CCH model of two-vessel occlusion (2VO), Niu et al. [94] demonstrated that the application of aerobic exercise during the chronic phase improved the restoration and expression of BDNF in the hippocampus at both the transcription and protein levels through the modulation of the NF-κB/miR-503 signaling pathway. This increase in BDNF was associated with profound improvements in cognitive ability, underlining the involvement of BDNF signaling in functional recovery. In support, Park et al. [95] observed that environmental enrichment (EE) significantly improved cognitive functions and the expression and upregulation of BDNF, phosphorylated CREB (pCREB), and VEGF in the hippocampus in addition to the enhancement of hippocampal angiogenesis compared to bilateral common carotid artery occlusion (BCCAO) models without EE. Sun et al. [96] also reported that EE restored reduced hippocampal BDNF and NR1 NMDA receptor expression, which was consistent with improved spatial and nonspatial memory compared to the same model. Furthermore, Hu et al. [97] demonstrated that postoperative intermittent fasting preserved hippocampal BDNF protein levels and postsynaptic density protein PSD-95, while attenuating oxidative stress, microglial activation, and neuroinflammation, resulting in significant improvements in memory and spatial learning during CCH. Choi et al. [98] demonstrated that treadmill exercise improved cognitive performance, promoted hippocampal neurogenesis, and increased expression of mature BDNF protein and pCREB, thus reinforcing the importance of activity-dependent NT signaling in cognitive resilience after chronic cerebral hypoperfusion. In accordance with those studies, Sakr et al. [99] demonstrated that dehydroepiandrosterone (DHEA) significantly improved working and reference memory, increased hippocampal BDNF levels and monoaminergic neurotransmitter concentrations, and protected hippocampal integrity after oclussion. Comparably, Wang and colleagues [100] found that andrographolide increased hippocampal BDNF and TrkB expression while simultaneously reducing astroglial activation, neuroinflammation, and apoptotic signaling, ultimately improving spatial learning and memory. Also, studies by Tian et al. [101] and Suna et al. [102] showed that dl-3-n-butylphthalide (NBP) significantly increased hippocampal BDNF expression during a phase of chronic hypoperfusion. Tian et al. highlighted SIRT1 activation as necessary for BDNF induction and cognitive improvement [101]. At the same time, Suna et al. showed that NBP-induced BDNF elevation was associated with enhanced cholinergic signaling, enhanced synaptic plasticity, and reduced oxidative stress and neuroinflammatory responses, which together facilitate functional recovery of cognitive performance [102].

Beyond just regulating BDNF levels, Wang and colleagues [103] showed that cornel iridoid glycoside increases the expression of BDNF and NGF in the hippocampus and cortex, along with their receptors TrkB and TrkA. This effect is combined with the activation of the PI3K/Akt/GSK-3β/CREB pathway and a reduction in hippocampal CA1 neuron loss. These molecular and structural changes led to noticeable improvements in learning and memory. Similarly, Zheng et al. [104] demonstrated that the injection of angelica restored the levels of reduced BDNF and NGF that resulted from hypoperfusion, and this restoration correlated with improved spatial learning. Extending neurotrophic support focused on NGF, Anastácio and colleagues [105] observed that resveratrol treatment increased hippocampal NGF expression in cases of chronic cerebral hypoperfusion. Interestingly, this increase happened gradually, becoming significant around 45 days after the occlusion, and was only seen in animals treated with resveratrol. This increase in NGF action was especially important in preventing the death of CA1 pyramidal cells and was accompanied by improvement in spatial working and reference memory, indicating that long-term neuroprotection can be induced by resveratrol through NGF-mediated trophic mechanisms. Further mechanistic research was carried out by Yin et al. [106], who showed that icariside II increased the levels of BDNF and TrkB in the hippocampus of rats, leading to increased levels of Akt and CREB phosphorylation alongside reduction of amyloidogenesis and improvement of cognitive abilities. Using a similar model, Niu et al. [107] showed that activation of the BDNF/TrkB and NRG1/ErbB4 pathways by epimedium flavonoids led to the preservation of synaptic structures as well as reduction of neuronal cell death alongside improvement of learning and memory abilities. In another study, Niu and team [108] found that these flavonoids also reduced white matter lesions and demyelination while boosting NT signaling in the corpus callosum, linking neurotrophic support to white matter health. Targeted delivery methods were explored by Li et al. [109], who used intranasal nano-inhalants of icariin to specifically enhance BDNF/TrkB signaling in the hippocampus and lower inflammatory cytokines, leading to improved cognition (Table 3). In addition to this, a study conducted by Zhang et al. [110] demonstrated that combining memantine with rosuvastatin augmented the levels of BDNF and VEGF in the hippocampus more potently than the drugs alone; this combination promoted the formation of new vessels, improvement of synaptic function, and facilitation of cognitive function.

Moreover, the neuromodulation techniques also appeared to support NT signaling pathways. Zhang et al. [111] demonstrated that repetitive transcranial magnetic stimulation helped restore hippocampal BDNF and VEGF levels, strengthened long-term potentiation, and improved spatial learning. Similarly, Zheng et al. [112] found that electroacupuncture helped reverse reductions in hippocampal BDNF, pCREB, and miR-132 caused by hypoperfusion, restoring synaptic plasticity and cognition through a PKA/CREB mechanism. In addition, a study conducted by Huang et al. [113] showed that the daily low-intensity pulsed ultrasound increased BDNF levels in the hippocampus, reduced neuronal damage and demyelination, and improved learning and memory.

In addition, Chen et al. [114] demonstrated that immunizing with glatiramer acetate helped restore hippocampal BDNF levels, normalized inflammation, protected cholinergic function, and improved synaptic plasticity and cognition. Epigenetic regulation has also been found to play a role, as demonstrated by Yao et al. [115], who found that overexpressing MeCP2 restored BDNF, TrkB, and CREB levels in the hippocampus and helped recover cognitive functions. Similar results were obtained by Lu et al. [116], indicating that the activation of the GABAB2 subunit restores BDNF, TrkB, and NCAM levels to normal and, consequently, normalizes Kir3 channels and diminishes anxiety behaviors (Table 3). Interestingly, Luo et al. [117] showed that baclofen improved working memory without changing BDNF levels, suggesting that some of its effects take place through region-specific or BDNF-independent pathways.

Furthermore, another study conducted by Jamhiri et al. [118] highlighted that chronic resveratrol treatment improved spatial learning and memory by lowering inhibitory molecules like hippocampal p75, Lingo-1, NgR1, RhoA, and ROCK2 while preserving neuron health, indicating that resveratrol might help by loosening the brakes on brain plasticity. Tiang et al. [119] found that compounds like α-mangostin from Garcinia mangostana improved memory, even without changing hippocampal BDNF at a subacute stage. Additionally, Han et al. [120] showed that epigallocatechin-3-gallate boosted cognitive function and reduced oxidative stress without affecting BDNF levels.

Additional studies also emphasize how important it is to support NTs in cases of chronic cerebral hypoperfusion. Shen et al. [121] have shown that melatonin and resveratrol alone or in combination increased BDNF in the hippocampus, reducing oxidative stress, inflammation, and cholinergic depletion. In a similar study, Al Dera et al. [122] investigated the effect of melatonin, which regulated SK channels and MAPK pathways and activated signals related to BDNF. Bhuvanendran et al. [123] reported that embelin helped restore hippocampal BDNF and CREB1, boosting synaptic plasticity and neurotransmitter balance. Furthermore, Jian et al. [124] reported that donepezil increased BDNF expression, while Wang et al. [125] found that boosting endocannabinoid signaling with URB597 restored hippocampal BDNF/TrkB levels and reduced neuronal death. In addition, Zhang et al. [126] reported that paeoniflorin not only reversed hippocampal BDNF loss induced by hypoperfusion but also has potential to relieve neural damage and improve cognitive function.

The mouse model of CCH with bilateral carotid artery stenosis (BCAS) consistently demonstrates that a reduction in neurotrophic support, synaptic integrity, and white matter homeostasis leads to cognitive impairment. Recent evidence suggests that a variety of interventions, from physical stimulation and pharmacological agents to epigenetic modulators, affect the BDNF/CREB signaling pathway to halt neurodegeneration and promote functional recovery.

Physical stimuli have also surfaced as significant inducers of neuroplasticity, such as high-frequency repetitive transcranial magnetic stimulation (HF-rTMS), which has demonstrated the ability to reduce cognitive deficits caused by CCH through increased expression of BDNF, MAP-2, and synapsin in the hippocampus. This neuroplastic change is accompanied by a strong anti-inflammatory response, characterized by the suppression of microglial (IBA-1) and astrocyte (GFAP) activation and a shift in the apoptotic balance, as indicated by the Bax/Bcl-2 ratio [127]. In addition, EA can activate similar pathways, but it primarily emphasizes the protection of white matter. EA treatment has also been shown to activate the ERK/CREB signaling cascade, leading to increased expression of TrkB and NGF (most significantly in the early phase of CCH) within white matter. This environment allows for the regeneration and maturation of the NG2^+^ progenitors into CC1^+^ oligodendrocytes, thus maintaining the structural integrity of the corpus callosum and hippocampus [128], as shown in Table 4.

As presented in Table 4, various pharmacological treatments [129,130,131,132] have also been investigated to assess their effects on the relationship between carotid occlusion and the neurotrophic system. The therapeutic effects of the combined treatment of aripiprazole and cilostazol have been shown to be superior to those of monotherapies, especially regarding the increased levels of mBDNF, as well as p-CREB in the hippocampal DG [129]. This suggests that the dual action of stabilizing the cAMP pathway and inhibiting phosphodiesterase promotes a more resilient neurotrophic environment. The role of nitric oxide (NO) has been investigated through the efficacy of nitric oxide-donating botanical blends (NOBMs). Orally administered NOBM preserves parvalbumin-positive inhibitory interneurons, which are highly susceptible to ischemic stress, while simultaneously enhancing cortical and hippocampal BDNF, thereby reducing neuronal loss and neuroinflammation [130]. Furthermore, pituitary adenylate cyclase-activating polypeptide (PACAP) has been shown to restore markers of synaptic plasticity, such as PSD-95, through a Sirt3-dependent mechanism. By increasing the expression of PAC1 receptors and Sirt3, PACAP effectively links mitochondrial metabolic health to increased BDNF [131]. In addition, ginsenoside Rd exerts neuroprotective effects by epigenetically modulating BDNF promoters, thereby increasing transcription within the prefrontal cortex and hippocampus. This targeted upregulation not only inhibits apoptosis but also shows the potential of small molecule ginsenosides to recalibrate genomic responses to chronic hypoperfusion [132].

Compared with the BCCAO animal model, unilateral carotid artery occlusion (UCCAO) induces a more moderate reduction in cerebral blood flow. This results in a neurobiological phenotype characterized by selective vulnerability of the hippocampus and cortex, with early disruption of NT signaling, including reduced BDNF levels, impaired activation of the receptor-mediated ERK-CREB pathways, and altered NT processing. Accordingly, unilateral chronic cerebral hypoperfusion (CCH) models provide a platform for examining NT-dependent mechanisms of cognitive decline and recovery under conditions that more closely resemble chronic carotid artery stenosis in humans than complete occlusion. In contrast, bilateral models produce more severe and widespread hypoperfusion with rapid structural damage, which may mask early changes in neurotrophic signaling. These differences suggest that unilateral CCH animal models are especially suited to identify NTs as early biomarkers and potential therapeutic targets in asymptomatic or mildly symptomatic carotid artery disease patients.

In aged rats subjected to UCCAO, a significant decrease in hippocampal BDNF protein levels was observed, along with a concomitant increase in proapoptotic signaling, inflammatory mediators, and markers of oxidative stress compared with healthy controls. Treatment with dimethyl fumarate (DMF) reversed the neurotrophic deficits by increasing hippocampal BDNF and the immediate early gene c-fos and activated the Nrf2 antioxidant pathway. Notably, restoration of BDNF by DMF was accompanied by a reduction in neuroinflammation, attenuation of apoptosis, and improvement in memory performance, placing NT recovery in a broader protective framework that integrates redox balance and inflammation control [133]. Similar NT suppression was observed in a study [134] of a mouse model of UCCAO, where CCH resulted in impaired recognition and memory, brain atrophy, increased acetylcholinesterase activity, and decreased hippocampal BDNF, p-ERK, and p-CREB compared to sham-operated controls, whereas administration of mumefural effectively restored BDNF expression and reactivated ERK/CREB signaling, consistent with normalization of cholinergic function and improved memory. These findings highlight that CCH-induced NT deficiency is closely linked to impaired neuroplasticity signaling and that pharmacological restoration of the ERK–CREB–BDNF axis is sufficient to restore cognitive function [134]. In accordance with those studies, Zhang et al. [135] demonstrated that mice with UCCAO showed reduced levels of BDNF and p-CREB in the hippocampus, associated with neuronal loss, white matter damage, and increased neuroinflammation compared with controls. EE restored BDNF and p-CREB expression, suppressed microglia and astroglia activation, and promoted microglia polarization toward an anti-inflammatory phenotype. Furthermore, after induction of CCH in the same model as in previous studies, EE preserved neurotrophic signaling and improved long-term cognitive and motor changes (Table 5).

Although most studies focus on total BDNF levels in CCH, emerging evidence suggests that changes in NT processing also contribute to outcomes. In a rat model of ischemic mild cognitive impairment induced by gradual bilateral carotid occlusion, an imbalance between proBDNF and mBDNF was observed, favoring the neurotoxic form of proBDNF despite overall synaptic damage and cognitive impairment, as shown in Table 5. Furthermore, treatment with Jiawei Kongsheng Zhenzhong Pill (JKZP) shifted BDNF processing toward its mature form by activating the S100A10/tPA pathway, thereby restoring synaptic structure and cognitive performance. These findings expand the NT framework beyond expression levels, highlighting that impaired extracellular processing of BDNF is an additional mechanism by which chronic hypoperfusion impairs synaptic integrity [136].

Collectively, these studies demonstrate that CCH consistently induces a state of NT deficiency and signaling dysfunction compared to healthy controls. This is characterized by reduced BDNF availability, impaired receptor-mediated intracellular pathways, and altered NT maturation. Furthermore, animal models reveal multiple NT regulatory pathways affected by hypoperfusion, including expression, downstream signaling, and processing, indicating the complex involvement of the neurotrophic system in vascular cognitive impairment.

Carotid artery disease rarely occurs in isolation in clinical practice. It most commonly develops in association with systemic comorbidities such as obesity, diabetes mellitus, and hypertension. These conditions affect cerebrovascular function, neuroinflammation, metabolic stress, and neuroplasticity, thereby altering the brain’s physiological response to reduced cerebral blood flow. Although animal experimental models of CCH, particularly BCCAO, have established a pattern of NT alterations and synaptic dysfunction, models that include metabolic or vascular comorbidities reveal a more heterogeneous and dynamic NT response. Changes in BDNF signaling and its downstream pathways appear to be modulated by systemic disease, suggesting that NT dysregulation in carotid occlusion reflects both the severity of hypoperfusion and the disruption of metabolic markers in the patient. Understanding how comorbid conditions modulate NT signaling under chronic hypoperfusion is therefore crucial for a clinical translational approach.

In a rat model of carotid occlusion, Kim and Kim [137] have shown that chronic cerebral hypoperfusion induces significant changes in hippocampal NT signaling that differ significantly by metabolic status. In non-obese CCH rats, hypoperfusion was associated with increased hippocampal BDNF expression and activation of downstream ERK and CREB signaling compared with sham-operated controls, despite significant cognitive impairment, suggesting an endogenous attempt to compensate for vascular insufficiency that was nevertheless insufficient to preserve cognitive function. In contrast, obese CHH rats showed significant suppression of the BDNF/ERK/CREB pathway compared to both sham and non-obese CCH groups, accompanied by more severe cognitive deficits. These findings suggest that obesity inhibits the compensatory increase in NT signaling, likely through metabolically related interference with NT response. Accordingly, the study highlights that NT dysregulation in vascular dementia is heterogeneous and strongly modulated by the systemic metabolic context, with obesity shifting the NT response from an undercompensatory state to an apparent decrease in BDNF and NGF (Table 6).

A study conducted by Kwon et al. [138] on diabetic rats exposed to CCH showed that cannabidiol treatment increased cerebral perfusion and reduced diabetes-related brain pathology. Cannabidiol partially prevented the decline in hippocampal BDNF levels, but not significantly, and reduced neuroinflammatory markers and improved performance on memory-related tests. Since those effects occurred without neurotrophic changes, this suggests that the preservation of cognitive function is selective [138]. Moreover, a study on diabetes-prone OLEFT rats with BCCAO [139] showed that the phosphodiesterase-3 inhibitor cilostazol significantly improved cognitive performance and increased hippocampal BDNF expression and CREB phosphorylation, suggesting restoration of NT-dependent transcriptional signaling, with reduced neuronal loss, supporting the concept that diabetes accelerates NT collapse in CCH, while pharmacological intervention can partially rescue this pathway [139].

Hypertension is another important modifier of NT response in CCH. In stroke-prone spontaneously hypertensive rats (SHRSP) subjected to BCAS, chronic inhibition of soluble epoxide hydrolase with TPPU prevented memory deficits and improved cerebrovascular endothelial function without altering cerebral perfusion or systemic blood pressure. Furthermore, TPPU increased hippocampal BDNF and duplocortin mRNA expression, suggesting improved neurotrophic support and neurogenesis. These data suggest that targeting vascular dysfunction may indirectly restore NT signaling in hypertensive CCH [140].

A study by Moon and colleagues [141] showed that repeated administration of platelet-rich plasma significantly improved cognitive performance, reduced hippocampal neuronal loss, and attenuated neuroinflammation in rats with vascular dementia induced by BCCAO in combination with hypovolemia. These effects were accompanied by restoration and further increase in hippocampal BDNF and TrkB expression, which were significantly reduced in untreated animals (Table 6). This study suggests that exogenous trophic support can effectively overcome deficits in NT levels caused by hypoperfusion.

These studies demonstrate that NT dysregulation in chronic cerebral hypoperfusion is highly context-dependent and strongly influenced by systemic comorbidities. While isolated hypoperfusion may elicit transient or compensatory NT activation, metabolic and vascular disease states can attenuate NT upregulation and reduce functional recovery. These findings underscore the need to incorporate comorbidity-relevant models when evaluating NT-targeted interventions and support NT signaling as a convergent, yet modifiable, pathway linking vascular pathology to cognitive decline.

The following animal studies predominantly use transient global cerebral ischemia or ischemia/reperfusion (IR) models, induced by BCCAO with subsequent reperfusion, rather than CCH models. Although these models differ in temporal dynamics and pathophysiological mechanisms, they are highly connected to clinical scenarios of sudden interruption of cerebral blood flow followed by reperfusion, such as cardiac arrest, perioperative hypotension, carotid endarterectomy or stenting, and global ischemic episodes during major vascular surgeries.

Using UCCAO as an ischemic preconditioning strategy before severe transient global ischemia, Kushwaha et al. [142] demonstrated that preconditioned mice exhibited lower mortality rates, improved motor and cognitive outcomes, and reduced astroglial and microglial activation (GFAP, IBA1) compared to non-preconditioned animals. Preconditioning upregulated markers of synaptic plasticity (PSD-95, synaptophysin) and BDNF and increased VEGF, suggesting that mild sustained hypoperfusion triggers adaptive plasticity and vascular changes that enhance resilience to subsequent acute ischemic injury [142], as presented in Table 7. Additionally, a study by Gonçalves et al. [143] showed that EE induced neuroprotection in the same ischemic model, improving short-term memory and reducing infarct size. However, hippocampal BDNF levels did not differ between groups; instead, the benefits associated with EE were aligned with reductions in IL-1β and modulation of astroglial reactivity (GFAP), suggesting that cognitive preservation may be driven by anti-inflammatory and glial mechanisms [143]. In accordance with these studies, the IR mice model involving 20 min of BCCAO with reperfusion and multistrain probiotic supplementation administered for three weeks before occlusion reduced hippocampal neuronal death and apoptosis in the hippocampus. Behaviorally, spatial learning and memory deficits were improved only at the highest dose (10^9^ CFU/day), whereas BDNF protein concentrations remained unchanged, indicating a BDNF-independent protective mechanism [144].

Contrary to these findings, a number of pharmacological studies show that BDNF restoration is linked to recovery and frequently accompanied by CREB-mediated transcription and decreased cell death. In a study conducted by Fan and colleagues [145], lithium chloride (LiCl) (2 or 5 mmol/kg, i.p.) improved spatial learning and memory assessed 31 days post-surgery, increased hippocampal neuron count, and upregulated hippocampal BDNF levels relative to controls. These effects were associated with decreased apoptosis (increased Bcl-2/Bax ratio) and elevated p-CREB, resulting in cognitive enhancement. Similarly, in BCCAO IR mice, huperzine A (0.2 mg/kg, oral) administered two days before surgery and continued for seven days post-surgery attenuated memory deficits and neuronal damage in both the cortex and hippocampus while augmenting BDNF and NGF levels and TGF-β1 (notably in the seven-day treatment group) and promoting MAPK/ERK1/2 phosphorylation [146]. A similar trend was seen in a study using this same model that treated the rats with daphnetin (40 mg/kg, i.p.), thereby improving the survival of hippocampus neurons and enhancing spatial memory. Daphnetin also improved outcomes related to BBB integrity (lower brain water content and increased claudin-5), elevated antioxidant capacity (increased SOD), decreased neuroinflammatory markers (NF-κB, IL-1β), and increased hippocampal BDNF protein, connecting NT recovery within a broader profile of changes [147].

Further insights are provided by studies focusing on receptor-linked plasticity pathways that connect behavioral rescue to NT preservation. A research study on a mouse IR model carried out by Xu et al. [148] found that activation of the sigma-1 receptor (σ1R) with PRE084 (1 mg/kg, ip) rescued learning and memory impairment (Table 7). The σ1R operates through a mechanism that prevents the reduction in hippocampal BDNF, as well as its related signal-transducing elements, including NR2A, CaMKIV, TORC1, and CREB. Additionally, PEAQX, a selective NR2A receptor antagonist, reduced these rescued behaviors, suggesting that NR2A, as a signaling mediator, is involved in the regulation of σ1R-induced BDNF expression. Extending this research, a different study by the same authors [149] showed that σ1R agonism with PRE084 (1 or 3 mg/kg, i.p.) or the non-selective agonist DTG (1 mg/kg, i.p.) enhanced cognitive performance, prevented reductions in BDNF and pTrkB, and upregulated NR2A/CaMKIV/TORC1. These effects were reversed by the σ1R antagonist BD1047, further confirming σ1R dependence and identifying a coherent NR2A/CaMKIV/TORC1/BDNF axis involved in post-ischemic cognitive recovery. An additional signaling mechanism, which was activated by using the phosphodiesterase type 2 inhibitor BAY 60-7550, resulted in decreased anxiety and increased cognitive function after treatment for 21 days following reperfusion. This was associated with increased hippocampal pCREB and BDNF and decreased neurodegeneration markers, which indicated increased NT-associated plasticity [150]. In the serotonergic domain, a study by Aguiar and colleagues [151] confirmed that chronic activation of postsynaptic 5-HT1A receptors with the agonist NLX-101 produced neurorestorative effects in global ischemia induced by BCCAO in mice. Initiated one week before ischemia and continued for 28 days, this regimen aimed to ensure sustained receptor engagement throughout injury and recovery. Chronic ischemia was associated with decreased levels of BDNF, synaptophysin, and PSD-95 in the hippocampus and prefrontal cortex, along with dendritic spine loss, elevated corticosterone, and cognitive and affective impairments. NLX-101 restored BDNF and synaptic protein levels in both regions, prevented dendritic degeneration, improved memory and despair-like behaviors, and inhibited ischemia-induced activation of the hypothalamic–pituitary–adrenal axis.

Beyond receptor agonism, several interventions suggest modulation of NTs via anti-inflammatory, antioxidant, and neurogenesis-supportive pathways. In a repeated global IR model of vascular dementia, fisetin [152] treatment improved cognitive performance and suppressed activation of NF-κB and the NLRP3 inflammasome (NLRP3/ASC/caspase-1), reducing IL-1β and IL-18 levels and upregulating NRF2/HO-1. Concomitantly, BDNF immunoreactivity increased, and apoptosis markers shifted towards cell survival (decreased BAX and increased Bcl-2), linking NT recovery and an integrated inflammation–redox–survival response (Table 7). In the same model, long-term administration of the BBB-penetrating peptide TAT-LBD-Ngn2 [153] enhanced spatial and contextual memory, increased hippocampal neurogenesis (BrdU^+^ and DCX^+^ cells in the dentate gyrus), and selectively increased BDNF protein levels, with NGF remaining unchanged, indicating that neurogenesis-linked recovery may be more closely associated with BDNF rather than NGF upregulation. A study of IR on rats conducted by Melindah et al. [154] demonstrated that intraperitoneal vitamin D administered for 10 days improved spatial memory after transient global ischemia and increased hippocampal NGF mRNA levels while reducing the senescence markers (p16 and p21), highlighting a possible non-BDNF trophic axis. Additionally, two studies [155,156] on the same rat model showed that remote limb ischemic postconditioning (RIPOC) can elicit NT-mediated protection during early reperfusion. In cerebral IR injury, RIPOC administered at the onset of reperfusion improved behavioral scores, enhanced antioxidant enzyme activity, reduced oxidative and inflammatory markers, and increased HO-1 and BDNF levels [155]. Notably, inhibition of HO-1 abolished neuroprotection, implicating an HO-1/BDNF-dependent mechanism. Also, another study [156] conducted by the same authors on the same model, in addition to the previous results, showed that RIPOC reduced the increased values of GSK as well as the levels of BNDF and CREB, which were induced by the rat model of IR, supporting a GSK-3β/CREB/BDNF pathway as a mediator of neurorestoration.

Studies conducted within the ischemia–reperfusion framework demonstrate that NT signaling, particularly BDNF, is a highly dynamic and intervention-sensitive component of post-ischemic brain recovery rather than a static marker of injury severity. Across pharmacological, neuromodulatory, conditioning, and behavioral interventions, the restoration or preservation of BDNF signaling often correlates with improvements in cognition, synaptic integrity, and neuronal survival, frequently through activation of CREB-dependent transcription. However, several studies also indicate that meaningful functional recovery may occur without detectable changes in BDNF levels, highlighting the contribution of alternative mechanisms.

Thus, the results of preclinical studies indicate a consistent dysregulation of the NT system in the context of chronic cerebral hypoperfusion and global cerebral ischemia associated with CAD (Table 4, Table 5, Table 6 and Table 7). Despite the differences in the animal models, therapeutic interventions, and experimental approaches, several common molecular mechanisms seem to converge throughout the presented preclinical research. Most of the effective therapeutic interventions were associated with the normalization of the BDNF/TrkB cascade, activation of subsequent intracellular signaling cascades such as PI3K/Akt and MAPK/ERK, and subsequent phosphorylation of the transcription factor CREB, which, in turn, supports neuronal survival, synaptic plasticity, and neurogenesis. In addition, several therapeutic approaches were associated with the attenuation of neuroinflammatory signaling pathways mediated by NF-κB, reduction of oxidative stress, and modulation of glial cell activation. These results suggest that different therapeutic methods in preclinical studies may achieve their neuroprotective effects through the common molecular mechanisms that regulate the NT system (mainly BDNF) and its associated signaling pathways in the context of CAD-related chronic cerebral hypoperfusion.

## 4. Conclusions

Taken together, the results obtained in clinical trials provide a clear conceptual framework in which chronic attenuation of BDNF is associated with maladaptive vascular remodeling. Contrary to this, acute increases in BDNF occur following restoration of cerebral blood flow, particularly when pharmacologically enhanced. This dual behavior supports the use of BDNF as both a chronic marker of vascular health and an acute biomarker of neurovascular recovery, with potential implications for patient stratification, therapeutic targeting, and monitoring in carotid artery disease. Indeed, the number of studies that evaluated the impact of NT system elements in clinical trials is insufficient, and, even more, focused almost only on BDNF alterations. Therefore, there is a need for bigger prospective clinical research due to the limited clinical evidence now available and the methodological limitations of prior studies.

On the other hand, animal models of chronic cerebral hypoperfusion, as the most frequent experimental design employed in preclinical studies, demonstrate that sustained reductions in cerebral blood flow disrupt NT signaling, leading to synaptic dysfunction, neuronal injury, and cognitive impairment. Obviously, the significantly higher number of preclinical studies (when compared to clinical trials) also resulted in the evaluation of a wider range of neurotrophic factors, including their receptors, and subsequent downstream pathways that demonstrate complex mechanisms for neurotrophic repair, which will enable researchers to translate preclinical data into clinically useful strategies for the treatment of neurotoxicity accompanied by CAD.

However, any rigorous analysis will lead to the conclusion that the obvious lack of standardization in methodology applied in both clinical trials and preclinical investigations, as well as the inconsistent selection of NT system elements included in evaluations, means that NT system elements must be considered as exploratory rather than as standard biomarkers in the broad field of neurotoxicity patterns accompanied by CAD.

## 5. Future Directions

According to data presented in this overview, it seems necessary that further clinical studies in this field should incorporate a greater number of NT system elements. This approach seems to be essential to comprehensively understand the mechanisms driving the NT element alterations that occur along with CAD and the development of novel, effective therapeutic options. Also, future research should focus on the timing of NT response and its impact on therapeutic intervention windows, thus making NT system element profiling more useful in investigations of CAD, including its severity, consequences, and therapeutic protocol effectiveness. Moreover, there is an urgent need to improve future research on using NT level measurements as potential biomarkers in CAD patients, including performing a larger scale of clinical studies, developing standardized protocols to measure NT levels, performing long-term studies to observe the changes in NT levels before and after revascularization, observing the changes in NT levels along with neuroimaging and cognitive studies, and observing the changes in different NT levels (BDNF, NGF, etc.) along with axonal injury markers such as NfL. Such approaches will be crucial for redefining NT system elements from exploratory to widely employed biomarkers and/or therapeutic targets in CAD.

## Figures and Tables

**Table 2:** NT system alterations in carotid artery diseases according to results obtained in clinical trials.

Reference/Pathological Entity	Treatment	NT System Alterations	Effect	Neuropsychiatric Outcome
[88] Hypertrophic remodeling of the carotid artery (*n* = 48)	Non-specific	↓ BDNF (1.9 ± 0.13 vs. 0.86 ± 0.1 ng/mL^−1^)	Low BDNF correlated with hypertrophic vascular remodeling	-
[89] Significant carotid stenosis (>70%) (*n* = 39)	Carotid artery stenting	↓ BDNF (6.37 ± 4.67 vs. 3.1 ± 3.1 ng/mL) ↑ NGF (21.48 ± 52.81 vs. 195.67 ± 495.34 pg/mL)	Post-stenting NT normalization indicates cerebral perfusion-dependent recovery	-
		Post-op (24 h): ↑ * BDNF (3.1 ± 3.1 vs. 4.99 ± 2.57 ng/mL) (n.c.) NGF (21.48 ± 52.81 vs. 94.92 ± 120.06 pg/mL)		
[90] Significant carotid stenosis (symptomatic > 50%, asymptomatic > 70%) (*n* = 80 + 80)	Carotid artery stenting	Post-op (24 h, 72 h): (n.c.) BDNF (~22 ± 10 vs. ~19 ± 10; ~23 ± 5 ng/mL)	↓ incidence of cerebral hyperperfusion syndrome	No adverse neurological outcomes
	Carotid artery stenting + DEX (0.1 mg/kg/h during and for 72 h after surgery)	Post-op (24 h, 72 h): ↑ ** BDNF (~20 ± 8 vs. ~28 ± 10, 32 ± 10 ng/mL)		
[91] Severe internal carotid artery stenosis (symptomatic > 50%, asymptomatic > 70%) (*n* = 24 + 25)	Endarterectomy	↑ * BDNF (after unclamping and 1 h post-op) (~470 ± 50 vs. ~560 ± 50; ~570 ± 50 pg/mL)	↓ inflammation	↑ cognitive metrics early post-surgery
	Endarterectomy + DEX (0.3 µg/kg loading dose, 10 min before anesthesia, 0.3 µg/kg^−1^/h^−1^ maintenance dose during surgery)	↑ * BDNF (after unclamping and 1 h post-op) (~460 ± 40 vs. ~580 ± 40; ~570 ± 40 pg/mL) ↑ ** BDNF (24 h post-op) (~450 ± 40 vs. ~570 ± 40 pg/mL)		
[92] Significant carotid stenosis (>70%) (*n* = 25 + 25)	Intermittent whole-body hypoxic preconditioning (7 days before surgery)	(n.c.) BDNF (~245 ± 40 vs. ~240 ± 45 pg/mL)	↓ postoperative neuronal injury markers	-

* denotes a significant change in values when compared to the pathological event, ** denotes a significant change in values when compared to the basal values, and *n* denotes the number of subjects employed in the study.

**Table 3:** NT system alteration in the animal model of chronic cerebral hypoperfusion induced by the permanent bilateral common carotid artery occlusion.

Reference/Model	Treatment	NT System Alterations		Effect	Clinical Outcome
		Model	Treatment		
[93] PBOCCA (rats)	-	↓ BDNF in hippocampus (14 and 28 days after surgery) (29.3 ± 3.1% and 40.1 ± 2.6% of sham)	-	↓ * neurotrophic support	No significant changes in locomotor activity ↓ * learning and memory
[94] 2VO (rats)	Exercise (30 min/d) from week 3 to week 7 after 2VO	↓ BDNF (~0.58 ± 0.1 of sham) (hippocampus)	↑ * BDNF (~0.88 ± 0.1 of sham) (hippocampus)	↓ * BDNF suppression, mediated via NF-κB/miR-503 signaling	↑ * learning and memory
[95] BCCAO (rats)	EE for 15 weeks	↓ BDNF (0.57 ± 0.07 vs. 0.48 ± 0.03) (hippocampus)	↑ * BDNF (0.48 ± 0.03 vs. 0.97 ± 0.05) (hippocampus)	↑ * pCREB and VEGF in the hippocampus	↑ * spatial and working memory
[96] 2VO (rats)	EE for 4 weeks	↓ BDNF (~0.35 ± 0.05 of sham) (hippocampus)	↑ * BDNF (~0.7 ± 0.05 of sham) (hippocampus)	↑ * synaptic plasticity	↑ * spatial and non-spatial memory performance
[97] 2VO (rats)	Postoperative intermittent fasting 1 week post-2VO, alternate-day feed deprivation for 8 weeks	↓ BDNF (~0.25 ± 0.05 of sham) (hippocampus)	↑ * BDNF (~0.7 ± 0.05 of sham) (hippocampus)	↓ * oxidative stress, reduced microglial activation	↑ * cognitive performance
[98] 2VO rat	Exercise (30 min/d) for 4 weeks starting 3 weeks after 2VO	(n.c.) BDNF (~145 ± 20% of sham) (hippocampus)	↑ * BDNF (~210 ± 15% of sham) (hippocampus)	↑ * hippocampal neurogenesis	↑ * spatial memory performance
[99] 2VO (rats)	DHEA (250 mg/kg/d, orally) for 7 days	↓ BDNF (~22 ± 2 vs. ~7 ± 2 pg/mL) (hippocampus)	↑ * BDNF (~7 ± 2 pg/mL vs. ~13 ± 2 pg/mL) (hippocampus)	↑ * central neurotransmitters (NA, DA, Ach)	↑ * working and reference memory
[100] 2VO (rats)	Andrographolide (10 mg/kg/d, i.p.) for 4 weeks	↓ BDNF (~0.35 ± 0.05 of sham) ↓ TrkB (~0.3 ± 0.05 of sham) (hippocampus)	↑ * BDNF (~0.7 ± 0.05 of sham) ↑ * TrkB (~0.6 ± 0.05 of sham) (hippocampus)	↓ * astroglial activation ↓ * neuroinflammation ↓ * neuronal apoptosis	↑ * spatial learning and memory
[101] BCCAO (rats)	NBP (30 and 60 mg/kg/d, orally) for 4 weeks	↓ BDNF (~0.3 ± 0.05 of sham) (hippocampus)	↑ * BDNF (~0.9 ± 0.1 of sham) (hippocampus)	↑ * neurotrophic signaling via the SIRT1/BDNF pathway	↑ * learning and memory
[102] 2VO (rats)	NBP (80 mg/kg/d, orally) for 2 weeks, starting 3 weeks after 2VO	(n.c.) BDNF (~1.1 ± 0.1 of sham) (hippocampus)	↑ ** BDNF (~1.7 ± 0.15 of sham) (hippocampus)	↑ * cholinergic system and synaptic plasticity ↓ * neuroinflammation ↓ * oxidative stress	↑ * learning and memory
[103] 2VO (rats)	Cornel iridoid glycoside (30–60–120 mg/kg/d, orally) for 3 months	↓ BDNF (~0.8 ± 0.05 of sham) ↓ NGF (~0.8 ± 0.05 of sham) ↓ TrkB (~0.8 ± 0.05 of sham) ↓ TrkA (~0.9 ± 0.05 of sham) (hippocampus and cortex)	↑ * BDNF (~1.1 ± 0.05 of sham) ↑ * NGF (~1.2 ± 0.05 of sham) ↑ * TrkB (~1.1 ± 0.05 of sham) ↑ * TrkA (~1.1 ± 0.05 of sham) (hippocampus and cortex)	↑ * PI3K/Akt/GSK-3β/CREB signaling ↑ * neuroplasticity	↑ * spatial learning and memory
[104] 2VO (rats)	Angelica (15 mL/kg/d i.v.) for 8 weeks	↓ BDNF (~4.1 ± 0.3 vs. ~1.4 ± 0.2) ↓ NGF (~1.5 ± 0.2 vs. ~0.35 ± 0.1) (hippocampus)	↑ * BDNF (~1.4 ± 0.2 vs. 2.7 ± 0.3) ↑ * NGF (~0.35 ± 0.1 vs. ~0.95 ± 0.1) (hippocampus)	BDNF and NGF levels positively correlated with the cognitive test	↑ * spatial learning and memory
[105] 2VO (rats)	Resveratrol (20 mg/kg/d, i.p.) for 7 days	↑ early NGF (~140% of sham) (hippocampus)	↑ early NGF (~132% of sham) ↑ * late NGF (~135% of sham) (hippocampus)	↓ * hippocampal CA1 pyramidal cell death	↑ * spatial working and reference memory
[106] BCCAO (rats)	Icariside II (8, 16 mg/kg/d orally) for 28 days starting 10 days after BCCAO	↓ BDNF (~0.7 ± 0.05 of sham) ↓ TrkB (~0.65 ± 0.05 of sham) (hippocampus)	↑ * BDNF (~0.95 ± 0.05 of sham) ↑ * TrkB (~0.9 ± 0.05 of sham) (hippocampus)	↓ * amyloidogenic processing	↑ * spatial learning and memory
[107] 2VO (rats)	Epimedium flavonoids (50, 100, 200 mg/kg/d, orally) for 12 weeks, starting 2 weeks after 2VO	↓ BDNF (~0.3 ± 0.05 of sham) (hippocampus)	↑ * BDNF (~1.1 ±0.1 of sham) (hippocampus)	↑ * PI3K/p-Akt/p-CREB signaling	↑ * learning and memory
[108] 2VO (rats)		↓ BDNF (~40 ± 10% of sham) ↓ TrkB (~55 ± 10% of sham) (corpus callosum)	↑ * BDNF (~95 ± 30% of sham) ↑ * TrkB (~95 ± 20% of sham) (corpus callosum)	↓ * Lingo-1/Fyn/ROCK signaling	↑ * spatial learning and memory
[109] BCCAO (rats)	Binary nano-inhalant icariin formulations	↓ BDNF (~0.9 ± 0.05 of sham) ↓ TrkB (~0.85 ± 0.05 of sham) (hippocampus)	↑ * BDNF (~1.2 ± 0.05 of sham) ↑ * TrkB (~1.25 ± 0.05 of sham) (hippocampus)	↑ * synaptic plasticity ↓ * inflammation	↑ * cognitive function
[110] 2VO (rats)	Memantine (10 mg/kg/d orally) and/or rosuvastatin (10 mg/kg/d orally) for 4 weeks	↓ BDNF (~60 ± 5% of sham) (hippocampus)	↑ * BDNF (~90 ± 5% of sham) (hippocampus)	↑ * hippocampal neovascularization ↑ * synaptic function	↑ * learning and memory
[111] 2VO (rats)	rTMS: 5 Hz, daily for 4 weeks	↓ BDNF (~14 ± 2% vs. ~6 ± 1% of expression) (hippocampus)	↑ * BDNF (~6 ± 1% vs. ~25 ± 2% of expression) (hippocampus)	↑ * angiogenesis ↑ * synaptic plasticity	↑ * synaptic plasticity (LTP) ↑ * spatial learning
[112] 2VO (rats)	EA for 7 days	↓ BDNF (~0.35 ± 0.02 vs. ~0.16 ± 0.02) (hippocampus)	↑ * BDNF (~0.16 ± 0.02 vs. ~0.27 ± 0.03) (hippocampus)	↑ * synaptic plasticity	↑ * spatial learning and memory
[113] BCCAO (rats)	Low-intensity pulsed ultrasound 5 min × 3 sessions per hemisphere daily for 2 weeks	↓ BDNF (~0.8 ± 0.05 of sham) (hippocampus)	↑ * BDNF (~1.1 ± 0.05 of sham) (hippocampus)	↓ * neuronal injury ↓ * demyelination	↑ * memory and learning
[114] 2VO (rats)	Glatiramer acetate (100 µg total, s.c.) 1st week: 2 times 2nd and 3rd weeks: 1 time	↓ BDNF (~1.31 ± 0.1 vs. ~0.75 ± 0.1) (hippocampus)	↑ * BDNF (~0.75 ± 0.1 vs. ~1.9 ± 0.1) (hippocampus)	↓ * glial activation normalized cytokine balance ↑ * cholinergic markers	↑ * spatial learning and memory
[115] 2VO (rats)	pGLV-mecp2 lentiviral plasmid, stereotactically in CA3 on the 3rd day after surgery	↓ BDNF (~0.6 ± 0.05 of sham) ↓ TrkB (~0.75 ± 0.05 of sham) (hippocampus)	↑ * BDNF (~1.05 ± 0.05 of sham) ↑ * TrkB (~1.05 ± 0.05 of sham) (hippocampus)	↑ * MeCP2	↑ * spatial learning and memory
[116] 2VO (rats)	Baclofen (25 mg/kg/d i.p.) for 23 days, starting 17 days after 2VO	↓ BDNF (~0.75 ± 0.05 of sham) ↓ TrkB (~1.1 ± 0.05 of sham) (hippocampus)	↑ * BDNF (~0.6 ± 0.05 of sham) ↑ * TrkB (~1.0 ± 0.05 of sham) (hippocampus)	Activation of GABA_B2_ restored BDNF signaling and normalized Kir3 channel surface expression	↓ * anxiety-like behavior
[117] 2VO (rats)	Baclofen (25 mg/kg/d, i.p.) for 3 weeks, starting 2 weeks after 2VO	(n.c.) BDNF (~1.0 ± 0.05 of sham) (PFC)	(n.c.) BDNF (~1.1 ± 0.05 of sham) (PFC)	↑ * HCN2	↑ * spatial working memory
[118] 2VO (rats)	Resveratrol (5 mg/kg/d, i.p.) for 35 days	↑ p75 (~1.4 ± 0.1 of sham) (hippocampus)	↓ * p75 (~0.9 ± 0.3 of sham) (hippocampus)	↑ * hippocampal neuronal integrity	↑ * spatial learning and memory
[119] VO2 (rats)	α-mangostin (50 mg/kg/d, orally) and XEFGM (100 mg/kg/d, orally) acute and 14-day sub-acute	↓ BDNF (~1.75 ± 0.25 vs. ~0.8 ± 0.15)	(n.c.) BDNF (~0.8 ± 0.15 vs. ~1.3 ± 0.25)	-	↑ * spatial learning and memory
[120] BCCAO (rats)	EGCG-single (25 mg/kg, i.v.) or multiple (50 mg/kg/d, i.p.) for 5 days starting 6 weeks after BCCAO	(n.c.) BDNF (~84.28 ± 20% of sham) (hippocampus)	(n.c.) BDNF (~91.46 ± 20% of sham) (hippocampus)	Modulation of VEGF and NMDA receptor subunits ↓ * oxidative stress	↑ * spatial learning and memory
[121] BCCAO (rats)	Melatonin (10 mg/kg/d) or resveratrol (20 mg/kg/d) or melatonin (5 mg/kg/d) + resveratrol (10 mg/kg/d) for 4 weeks	↓ BDNF (~25.0 ± 3.0 vs. ~9.5 ± 1.0 ng/mL) (hippocampus)	↑ * BDNF (~9.5 ± 1.0 vs. ~20.0 ± 0.1 ng/mL) (hippocampus)	↓ * oxidative stress ↓ * inflammatory markers ↓ * AChE activity	↑ * spatial learning and memory
[122] 2VO (rats)	Melatonin (10 mg/kg/d, i.p.) for 1 week	↓ BDNF (~0.22 ± 0.03 vs. ~0.09 ± 0.01) (hippocampus)	↑ * BDNF (~0.09 ± 0.01 vs. ~0.21 ± 0.02) (hippocampus)	↓ * SK1, SK2, SK3 channels ↓ * inflammation ↑ * antioxidant status	↑ * spatial learning and memory
[123] BCCAO (rats)	Embelin (0.3, 0.6, 1.2 mg/kg/d, i.p.) for 5 days, starting 2 weeks after BCCAO	↓ BDNF (~5.5 ± 0.6 vs. ~2.2 ± 0.5) (hippocampus)	↑ * BDNF (~2.2 ± 0.5 vs. ~6.4 ± 1.1) (hippocampus)	↑ * synaptic plasticity ↓ * oxidative stress ↓ * pro-inflammatory signaling ↑ * neurotransmitter balance	↑ * spatial learning and memory
[124] BCCAO (rats)	Donepezil (10 mg/kg/d, orally) for 3 weeks, starting 2 weeks after BCCAO	↓ BDNF (~65 ± 6% of sham) (cortex and hippocampus)	↑ * BDNF (~95 ± 5% of sham) (cortex and hippocampus)	↑ * neurotrophic signaling	↑ * spatial learning and memory
[125] BCCAO (rats)	URB597 (0.3 mg/kg/d, i.p.) for 8 weeks	↓ BDNF (~0.93 ± 0.04 vs. ~0.7 ± 0.05) ↓ TrkB (~0.74 ± 0.03 vs. ~0.38 ± 0.04) (hippocampus)	↑ * BDNF (~0.7 ± 0.05 vs. ~0.83 ± 0.06) ↑ * TrkB (~0.38 ± 0.04 vs. ~0.48 ± 0.05) (hippocampus)	↓ * apoptosis	↑ * spatial learning and memory
[126] BCCAO (rats)	Paeoniflorin (20 mg/kg/d or 40 mg/kg/d, orally) for 4 weeks	↓ BDNF (~0.9 ± 0.3 vs. ~0.4 ± 0.1) (hippocampus)	↑ * BDNF (~0.4 ± 0.1 vs. ~0.7 ± 0.1) (hippocampus)	↓ * neuronal damage ↓ * apoptosis	↑ * spatial learning and memory

* denotes a significant change in values when compared to the pathological event and ** denotes a significant change in values when compared to the basal values.

**Table 4:** NT system alteration in the animal model of chronic cerebral hypoperfusion induced by the permanent bilateral carotid artery stenosis.

Reference/Model	Treatment	NT System Alterations		Effect	Clinical Outcome
		Model	Treatment		
[127] BCAS (mice)	HF-rTMS daily high-frequency stimulation for 2 weeks	↓ BDNF (~0.55 ± 0.05 of sham) (hippocampus)	↑ * BDNF (~0.85 ± 0.1 of sham) (hippocampus)	↓ * neuronal apoptosis ↓ * microglial activation ↓ * inflammation	↑ * memory
[128] BCAS (mice)	EA	After 14 days: ↑ NT4 (~33 ± 3 vs. ~39 ± 3 cells) (n.c.) TrkB (~7.5 ± 2 vs. ~12 ± 3 cells) After 28 days: ↑ NT4 (~32 ± 3 vs. ~56 ± 7 cells) ↑ TrkB (~4 ± 1 vs. ~28 ± 4 cells)	After 14 days: ↑ ** NT4 (~33 ± 3 vs. ~58 ± 10 cells) ↑ ** TrkB (~7.5 ± 2 vs. ~26.5 ± 2 cells) After 28 days: ↑ ** NT4 (~32 ± 3 vs. ~62 ± 8 cells) ↑ ** TrkB (~4 ± 1 vs. ~22 ± 2 cells)	↑ * markers of oligodendrocyte regeneration and maturation	↑ * spatial learning and memory
[129] BCAS (mice)	Cilostazol (20 mg/kg/d orally) + aripiprazole (0.5 mg/kg/d orally) for 3 weeks	(n.c.) mBDNF (~50 ± 6 vs. ~45 ± 4 cells) (DG)	↑ ** mBDNF (~50 ± 6 vs. ~150 ± 35 cells) (DG)	↑ * p-CREB ↓ neuronal apoptosis	↑ * spatial learning and memory
[130] BCAS (mice)	NOBM (0.1 mL orally) twice daily for 4 weeks	↓ BDNF (~0.33 ± 0.03 of sham) (hippocampus and cortex)	↑ * BDNF (~0.42 ± 0.05 of sham) (hippocampus and cortex)	↓ * neuronal loss ↓ * neuroinflammation preserved parvalbumin interneurons	↑ * spatial memory and place recognition performance
[131] BCAS (mice)	PACAP (3 μL per nostril) 5 times per week for 4 weeks, starting 30 days after BCAS	↓ BDNF (~0.7 ± 0.15 of sham) (hippocampus and cortex)	↑ * BDNF (~1.3 ± 0.3 of BCAS) (hippocampus and cortex)	↑ * synaptic plasticity	↑ * spatial learning and memory
[132] BCAS (mice)	Ginsenoside Rd (10 or 30 mg/kg/d i.p.) for 3 weeks	↓ BDNF (~0.45 ± 0.15 of sham) (hippocampus and PFC)	↑ * BDNF (~0.88 ± 0.12 of sham) (hippocampus and PFC)	↑ * neuronal survival ↓ * apoptosis ↑ * neuroprotection	↑ * cognitive functions

* denotes a significant change in values when compared to the pathological event and ** denotes a significant change in values when compared to the basal values.

**Table 5:** NT system alteration in the animal model of chronic cerebral hypoperfusion induced by the permanent unilateral carotid artery stenosis.

Reference/Model	Treatment	NT System Alterations		Effect	Clinical Outcome
		Model	Treatment		
[133] UCCAO (rats)	DMF (100 mg/kg, orally) 3 times/week for 4 weeks	↓ BDNF (2.4 ± 0.3 vs. 0.6 ± 0.6 ng/g^−1^) (hippocampus)	↑ * BDNF (0.6 ± 0.6 vs. 3.0 ± 0.3 ng/g^−1^) (hippocampus)	↓ * inflammation ↓ * apoptosis	↑ * cognitive functions
[134] UCCAO (mice)	Mumefural (40 mg/kg/d, orally) for 8 weeks	↓ BDNF (~55 ± 8% of sham) (n.c.) pTrkB/TrkB (~115 ± 10% of sham) (hippocampus)	↑ * BDNF (~105 ± 10% of sham) (n.c.) pTrkB/TrkB (~100 ± 8% of sham) (hippocampus)	↓ * apoptosis ↓ * AChE activity	↑ * cognitive functions
[135] UCCAO (mice)	EE for 3 weeks	↓ BDNF (~0.88 ± 0.05 vs. ~0.36 ± 0.07) (hippocampus)	↑ * BDNF (~0.36 ± 0.07 vs. ~0.54 ± 0.05) (hippocampus)	↓ * neuronal loss ↓ * inflammation	↑ * cognitive functions
[136] BCCAO (rats)	JKZP (56.7 g/kg orally) 5 times in 60 days	↑ proBDNF/mBDNF ratio (~1.1 ± 0.2 vs. 6.1 ± 0.9) ↑ P75^NTR^ (~3.7 ± 0.2 of sham) ↓ TrkB (~0.75 ± 0.1 of sham)	↓ * proBDNF/mBDNF ratio (~6.1 ± 0.9 vs. 4.0 ± 0.5) ↓ * P75^NTR^ (~3.0 ± 0.5 of sham) ↑ * TrkB (~1.05 ± 0.1 of sham)	↑ * neuroplasticity ↑ * dendritic spine density	↑ * cognitive functions

* denotes a significant change in values when compared to the pathological event.

**Table 6:** NT system alteration in the animal model of chronic cerebral hypoperfusion combined with other models of diseases.

Reference/Model	Treatment	NT System Alterations		Effect	Clinical Outcome
		Model	Treatment		
[137] BCCAO + obesity (rats)	High-fat diet for 8 weeks before BCCAO	↑ BDNF (~0.45 ± 0.04 vs. 0.80 ± 0.06) (hippocampus)	↓ * BDNF (~0.80 ± 0.06 vs. 0.30 ± 0.05) (hippocampus)	↓ * synaptic plasticity	↓ * spatial and working memory in obesity
[138] VA + left ICA + right ICA + diabetes (rats)	Cannabidiol (10 mg/kg/d) for 30 days	↓ BDNF (~1.15 ± 0.15 vs. 0.65 ± 0.07) (hippocampus)	(n.c.) BDNF (~0.65 ± 0.07 vs. 0.85 ± 0.10) (hippocampus)	↓ * markers of neuroinflammation	↑ * cognitive performance
[139] OLETF + BCCAO (rats)	Cilostazol (50 mg/kg/d, orally) for 2 weeks	↓ BDNF (~70 ± 5% of sham) (hippocampus)	↑ * BDNF (~75 ± 5% of sham) (hippocampus)	↑ * pCREB ↑ * survival of hippocampal neurons	↑ * spatial learning and memory
[140] SHRSP + BCAS (rats)	TPPU (3 mg/kg/d, orally) for 8 weeks	-	↑ * BDNF (~0.9 ± 0.15 vs. 1.55 ± 0.12)	↑ * endothelial function, altered oxidative stress, and inflammation	↑ * cognitive performance
[141] (BCCAO/H) (rats)	Platelet-rich plasma (500 µL, i.p.) on postoperative days 0, 2, 4, 6, and 8	↓ BDNF (~0.45 ± 0.1 of sham) ↓ TrkB (~0.5 ± 0.1 of sham) (hippocampus)	↑ * BDNF (~4.4 ± 0.3 of sham) ↑ * TrkB (~0.5 ± 0.1 of sham) (hippocampus)	↑ * viable neurons ↓ * neuroinflammation	↑ * spatial and learning memory

* denotes a significant change in values when compared to the pathological event.

**Table 7:** NT system alteration in an animal model of global cerebral ischemia induced by transient bilateral common carotid artery occlusion.

Reference/Model	Treatment	NT System Alterations		Effect	Clinical Outcome
		Model	Treatment		
[142] BCCAO (mice)	UCCAO for 10–12 weeks before BCCAO	-	↑ * BDNF (~1.55 ± 0.1 of BCCAO)	↓ * glial activation ↑ * neuronal resilience	↑ * motor and cognitive performance
[143] BCCAO mice	EE for 5 weeks before BCCAO	(n.c.) BDNF (~90 ± 15% of sham)	(n.c.) BDNF (~130 ± 20% of sham)	↓ * IL-1β ↑ * glial activation	↓ * short-term memory deficit
[144] BCCAO (mice)	Multistrain probiotics (10^7^, 10^8^, 10^9^ CFU/d, orally) for 3 weeks before BCCAO	(n.c.) BDNF (~0.95 ± 0.15 vs. 1.2 ± 0.05) (hippocampus)	(n.c.) BDNF (~0.95 ± 0.15 vs. 1.10 ± 0.1) (hippocampus)	↓ * apoptosis	↑ * spatial learning/memory at 10^9^ CFU/day
[145] BCCAO (mice)	LiCl (2 mmol/kg/d or 5 mmol/kg/d, i.p.) before (7 days) or after (28 days) BCCAO	↓ BDNF (~0.48 ± 0.05 of sham) (hippocampus)	↑ * BDNF (~0.85 ± 0.05 of sham) (hippocampus)	↓ * apoptosis ↑ * p-CREB	↑ * spatial learning and memory
[146] BCCAO (mice)	Huperzine A (0.2 mg/kg/d, orally) 2 days before and 7 days after BCCAO	(n.c.) BDNF (~110 ± 5% of sham) (n.c.) NGF (~105 ± 5% of sham) (hippocampus and cortex)	↑ ** BDNF (~145 ± 15% of sham) ↑ ** NGF (~125 ± 10% of sham) (hippocampus and cortex)	↑ * TGF-β1 ↑ * MAPK/ERK1/2 phosphorylation	↑ * spatial memory
[147] BCCAO (mice)	Daphnetin (40 mg/kg i.p.) immediately after BCCAO	↓ BDNF (~35 ± 4% vs. ~13 ± 3%) (hippocampus)	↑ * BDNF (~13 ± 3% vs. ~23 ± 5%) (hippocampus)	↑ * neuronal survival ↓ * inflammation preserved BBB	↑ * spatial memory
[148] BCCAO (mice)	PRE084 (1 mg/kg/d, i.p.) for 3 weeks	↓ NT-3 (~0.55 ± 0.05 of sham) ↓ BDNF (~0.58 ± 0.1 of sham) (hippocampus)	↓ ** NT-3 (~0.60 ± 0.05 of sham) ↑ * BDNF (~0.92 ± 0.10 of sham) (hippocampus)	↑ * neuroplasticity	↑ * learning and memory (NR2A antagonist reversed effects)
[149] BCCAO (mice)	PRE084 (1–3 mg/kg/d, i.p) or DTG (1 mg/kg/d, i.p) for 3 weeks	↓ BDNF (~0.55 ± 0.05 of sham) ↓ pTrkB/TrkB (~0.60 ± 0.05 of sham) (hippocampus)	↑ * BDNF (~0.90 ± 0.10 of sham) ↑ * pTrkB/TrkB (~0.95 ± 0.10 of sham) (hippocampus)	↑ * NR2A-CaMKIV-TORC1	↑ * spatial learning and memory
[150] BCCAO (mice)	BAY 60-7550 (1 mg/kg/d, orally) for 3 weeks	↓ BDNF (~0.22 ± 0.03 vs. ~0.10 ± 0.02) (hippocampus)	↑ * BDNF (~0.10 ± 0.02 vs. ~0.17 ± 0.05) (hippocampus)	↓ * neurodegeneration	↓ * anxiety-like behavior ↑ * cognition
[151] BCCAO (mice)	NLX-101 (0.32 mg/kg/d, i.p.) for 4 weeks	↓ BDNF (~41.22 ± 6 vs. ~23 ± 4 pg/μg) (hippocampus and PFC)	↑ * BDNF (~23 ± 4 vs. ~44 ± 7 pg/μg) (hippocampus and PFC)	↓ * corticosterone ↑ * synaptic plasticity	↑ * spatial memory ↓ * despair-like behaviors
[152] BCCAO (mice)	Fisetin (40 mg/kg/d, orally) for 15 days	↓ BDNF (hippocampus) IF technique	↑ * BDNF (hippocampus) IF technique	↓ * pro-inflammatory ↑ antioxidant capacity ↓ * apoptosis	↑ * spatial learning and memory
[153] BCCAO (mice)	TAT-LBD-Ngn2 (250 μg/kg/d, i.p.) for 14–28 days	-	(n.c.) NGF (~0.97 ± 0.04 vs. ~1.05 ± 0.05) ↑ * BDNF (~1.05 ± 0.05 vs. ~1.85 ± 0.05) (hippocampus)	↑ * neurogenesis	↑ * hippocampal neurogenesis and memory
[154] BCCAO (rats)	Vitamin D (0.125 µg/kg/d or 0.5 µg/kg/d, i.p.) for 10 days	↓ NGF (~1.6 ± 0.3 vs. ~0.85 ± 0.2) (hippocampus)	↑ * NGF (~0.85 ± 0.2 vs. ~1.7 ± 0.3) (hippocampus)	↓ * cellular senescence	↑ * spatial memory
[155,156] BCCAO (rats)	RIPOC, 3 cycles of 10 min hindlimb ischemia/reperfusion at the onset of cerebral reperfusion	↓ BDNF (~120 ± 8 vs. ~65 ± 7 ng/mg)	↑ * BDNF (~65 ± 7 vs. ~102 ± 7 ng/mg)	↓ * oxidative stress ↓ * inflammation ↓ * AChE activity	↑ * neurological and cognitive performance

* denotes a significant change in values when compared to the pathological event and ** denotes a significant change in values when compared to the basal values.

## Data Availability

No new data were created or analyzed in this study. Data sharing is not applicable to this article.

## Abbreviations

The following abbreviations are used in this manuscript:

CAD	carotid artery disease
CAS	carotid artery stenosis
CTA	CT angiography
MRA	magnetic resonance angiography
NASCET	North American Symptomatic Carotid Endarterectomy Trial
ICA	internal carotid artery
ECST	European Carotid Surgery Trial
CCH	chronic cerebral hypoperfusion
TIA	transient ischemic attack
AHN	adult hippocampal neurogenesis
CEA	endarterectomy
CAS	carotid artery stenting
SVD	small-vessel disease
BBB	blood–brain barrier
NT	neurotrophin
BDNF	brain-derived neurotrophic factor
NGF	nerve growth factor
NT-3	neurotrophin-3
NT-4	neurotrophin-4
Trk	tropomyosin-related kinase
p75NTR	p75 NT receptor
NfL	neurofilament light chain
pro-NTs	proneurotrophins
SCFAs	short-chain fatty acids
MS	multiple sclerosis
CVS	cardiovascular system
SMCs	smooth muscle cells
ECs	endothelial cells
VSMCs	vascular smooth muscle cells
MI	myocardial infarction
CVD	cardiovascular disease
BMECs	brain microvascular endothelial cells
VEGF	vascular endothelial growth factor
MDD	major depressive disorder
AD	Alzheimer’s disease
mPFC	medial prefrontal cortex
PSD	post-stroke depression
DEX	dexmedetomidine
PBOCCA	permanent bilateral carotid artery occlusion
2VO	two-vessel occlusion
EE	environmental enrichment
pCREB	phosphorylated CREB
BCCAO	bilateral common carotid artery occlusion
DHEA	dehydroepiandrosterone
NBP	dl-3-n-butylphthalide
BCAS	bilateral carotid artery stenosis
EA	electroacupuncture
HF-rTMS	high-frequency repetitive transcranial magnetic stimulation
NOBM	oxide-donating botanical blend
PACAP	adenylate cyclase-activating polypeptide
UCCAO	unilateral carotid artery occlusion
JKZP	Jiawei Kongsheng Zhenzhong Pill
SHRSP	stroke-prone spontaneously hypertensive rats
IR	ischemia/reperfusion
σ1R	sigma-1 receptor
RIPOC	remote limb ischemic postconditioning
